# A novel VOC breath tracer method to evaluate indoor respiratory exposures in the near- and far-fields

**DOI:** 10.21203/rs.3.rs-1437107/v2

**Published:** 2022-03-11

**Authors:** Hooman Parhizkar, Mark Fretz, Aurélie Laguerre, Jason Stenson, Richard L. Corsi, Kevin G. Van Den Wymelenberg, Elliott T. Gall

**Affiliations:** Institute for Health in the Built Environment, University of Oregon, Eugene, Oregon; Institute for Health in the Built Environment, University of Oregon, Portland, Oregon; Department of Mechanical and Materials Engineering, Portland State University, Portland, Oregon; Energy Studies in Building Laboratories, University of Oregon, Portland, Oregon; College of Engineering, UC Davis, Davis, California; Institute for Health in the Built Environment, University of Oregon, Eugene, Oregon; Department of Mechanical and Materials Engineering, Portland State University, Portland, Oregon

**Keywords:** Bioaerosol, COVID-19, Healthy Buildings, Social Distancing, Risk, Infectious Disease, PTR-ToF-MS, Near-field, Far-field

## Abstract

Several studies suggest that far-field transmission (> 6 ft) explains the significant number of COVID-19 superspreading outbreaks. Therefore, quantitative evaluation of near- and far-field exposure to emissions from a source is key to better understanding human-to-human airborne infectious disease transmission and associated risks. In this study, we used an environmentally-controlled chamber to measure volatile organic compounds (VOCs) released from a healthy participant who consumed breath mints, which contained unique tracer compounds. Tracer measurements were made at 2.5 ft, 5 ft, 7.5 ft from the participant, as well as in the exhaust plenum of the chamber. We observed that 2.5 ft trials had substantially (~36–44%) higher concentrations than other distances during the first 20 minutes of experiments, highlighting the importance of the near-field relative to the far-field before virus-laden respiratory aerosol plumes are continuously mixed into the far-field. However, for the conditions studied, the concentrations of human-sourced tracers after 20 minutes and approaching the end of the 60-minute trials at 2.5 ft, 5 ft, and 7.5 ft were only ~18%, ~11%, and ~7.5% higher than volume-averaged concentrations, respectively. Our findings highlight the importance of far-field transmission of airborne pathogens including SARS-CoV-2, which need to be considered in public health decision making.

## Introduction

The spread of COVID-19 has caused extensive damage to the lives of millions of people worldwide. Severe acute respiratory syndrome coronavirus 2 (SARS-CoV-2), the causative agent of COVID-19, is transmitted from human to human via bioaerosols particles that are released during respiratory activities such as breathing, talking, singing, and coughing.^1–3^

Substantial evidence supports that indoor spaces are hotspots where COVID-19 transmits beyond 6 ft of the source emitter.^3–7^ Furthermore, epidemiological studies, public health, and engineering risk assessment models indicate that the majority of well-documented superspreading outbreaks can be explained by bioaerosols beyond 6 ft from the source emitter.^8–10^ Therefore, quantifying the degree of exposure to bioaerosols according to distance from the emitting source is critical to more accurately characterize disease transmission risk, to determine the most effective risk reduction strategies such as ventilation, filtration, and spatial distancing, and ultimately to reduce disease transmission.

A well-mixed air space is a conventional assumption that has been used in most studies of indoor air pollution and infectious disease transmission modeling.^11^ For a well-mixed condition, indoor air contaminants, including virus laden aerosol particles, are assumed to be uniformly distributed by appropriate ventilation, interior mixing fans, buoyancy driven flows, and infiltration, immediately after being emitted from the source.^12^ However, thermal stratification, inadequate ventilation, and some environmental conditions can cause a non-uniform distribution of bio-aerosols in indoor spaces,^13,14^ where the probability of susceptible occupants inhaling virus-laden aerosol particles will rely, at least to some extent, on the distance from the source emitter.

Few studies have considered the importance of spatial parameters such as room height into measurements of indoor pollutants.^15–18^ A study of temporal and spatial scales suggests that chemical compounds as well as particles in the range of 1–10 μm with persistent residence time exhibit spatial gradients that are significantly controlled by ventilation rates.^19^ Additionally, controlled experiments on participants who were diagnosed with COVID-19 were used to study the abundance of SARS-CoV-2 viral RNA copies in room aerosols. The authors found that the near-field was associated with a higher number of virus RNA copies, and statistically higher carbon dioxide (CO_2_), and particle counts of 0.3 μm – 2.5 μm than in the far-field.^3^ Differences between near-field and far-field were also examined through CO_2_ and particles with patients receiving high-flow nasal cannula therapy (HFNC), where the CO_2_ concentration was statistically higher at a distance 0.5 m (~1.6 ft) from the source emitter compared to background levels.^20^

The goal of the present study is to better characterize the impact of distance from source on distribution of exhaled bioaerosols in an indoor environment. A novel trace gas approach is proposed where a participant consumed breath mints and released known compounds in exhaled breath.

## Methodology

### Methodology background

A previous study has shown that chewing peppermint flavored gum is associated with release of unique volatile organic compounds (VOCs) such as menthone and menthol, with source strength dependent on oral activity and chewing frequency.^21^ Real-time measurements of VOCs can provide useful information for studying pollutant dynamics of indoor environments.^22^ We used proton transfer reaction - time of flight - mass spectrometry (PTR-ToF-MS) to measure volatile organic compounds (VOCs) associated with breath mints across a mass range of 17–490 amu with 1 second time resolution. The principles of the PTR-ToF-MS measurements have been well-described previously.^23–25^ This approach allows for a real-time measurement of VOCs with a proton affinity greater than that of H_2_O. In theory, ionization is soft, allowing for little fragmentation, and compound identification can be made by observation of the [M+H]+ ion (i.e., molecular mass + the mass of the transferred proton). Our study used breath mints instead of chewing gum to trace three specific compounds (menthone, monoterpenes, and menthol).

### Participant Recruitment

Human subjects protocols were reviewed and approved by the University of Oregon Institutional Review Board (IRB) (Protocol #20210509). One human subject participated in this study. The participant was instructed to:

1. not use cologne or body sprays during the day preceding and during the study period;
2. wear clothes that were not recently washed with detergents;
3. follow a consistent diet during the course of three data collection days;
4. maintain a constant breath mint consuming rate during all trials.

### Climate chamber

**Figure 1:**
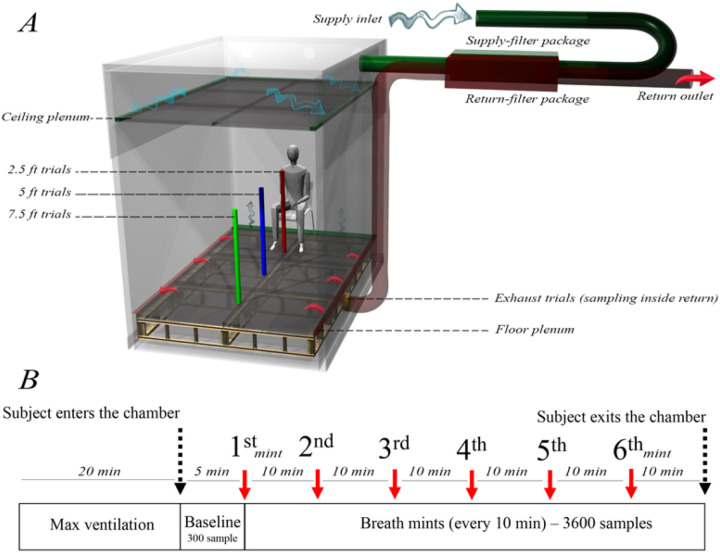
Experimental setup, A) climate chamber, airflow distribution, as well as sampling location for each unique trial (modeled in Rhinoceros software), B) experimental procedure and the number of breath mints consumed by the participant for each trial.

Experiments were conducted at the Energy Studies in Buildings Laboratory, Portland, OR, USA, using a custom environmentally-controlled climate chamber with an interior volume of 27 m^3^ (Figure 1A). Filtered air was supplied through a ceiling plenum and exhausted through a floor plenum. Air was exchanged at ~3 air changes per hour (ACH) during test periods and flushed at > 20 ACH for a minimum of 20 minutes between trial periods. We observed the concentration of breath tracers during the experiment as a distinct VOC that is associated with breath mints to confirm the removal of previous residuals before the beginning of each trial.

Ambient indoor air was supplied through a MERV-13 prefilter and high-flow activated carbon filters (Air Box 4 Stealth; AirBox Filters, Laval, Quebec, CA) and exhausted through an identical filtration system (shown as supply filter package and return filter package in Figure 1A). Air exchange rates were monitored throughout the experiments by balancing supply and exhaust air velocities measured at center-of-duct locations using a thermal anemometer and multi-function ventilation meter (#964 and #9565-P, respectively; TSI Incorporated, Shoreview, MN, USA).

Each trial began with adjustment of the climate chamber’s ventilation rate to the maximum value (20 ACH) for a minimum duration of 20 minutes without the presence of the participant in order to evacuate detectable residuals of prior trials (Figure 1B). We monitored the concentration of menthone to assure it reached a negligible steady-state background concentration. Next, the participant was instructed to enter the chamber, sit in a chair, and breathe normally for five minutes without consuming any breath mints. These 5-minute periods provided a baseline reference for each trial and were included in the study protocol to identify certain compounds that are exclusively associated with natural human breath and not breath mint flavoring, and to additionally provide a baseline to observe any exhaled compounds that may have remained in the participant’s mouth from previous trials. After 5 minutes, the participant was visually informed to begin consuming one breath mint every 10 minutes (Figure 1B), resulting in 6 breath mints consumed during each 1-hour trial (minutes 0, 10, 20, 30, 40, and 50). All breath mints were carried into the chamber by the participant in an air sealed plastic bag. To keep emissions relatively constant, the participant was instructed to remain silent and minimize body movement during the entire course of study. The participant also took care to maintain a resting activity level between trials to avoid emission irregularities while inside the chamber during the trials.

A summary of all trials conducted in this study is presented in Table 1. We used a single sampling line attached to a portable tripod and moved the probe to designated spots on the floor, measuring 2.5 ft, 5 ft and 7.5 ft from the participant’s mouth (Table 1, Trials A-C). Additionally, we placed another sampling line of equal length inside the floor plenum exhaust duct (called exhaust trials) to measure exhaust air as a “well-mixed” approximation of the volume -averaged concentration (Table 1, Trial D).

In addition to trials A-D (Table 1) within the climate chamber (Figure 1A), we conducted two other experiments to confirm the presence of unique tracer compounds associated with the exhaled breath of the participant consuming breath mints (Trials E&F, Table 1). In trial E, we placed one single breath mint in the headspace of a 250 mL glass container for ~1 minute and monitored the concentration of VOCs over a 20-minutes period. In trial F, the participant was instructed to consume one breath mint while breathing normally into the same 250 mL glass container for ~1 minute. Similar to trial E (Table 1), we monitored the concentration of VOCs over a 20-minute period. For trials E and F (Table 1), PTR-ToF-MS sampled at a flowrate of ~100 cc/min during both experiments and three minutes of background (BCK) measurements are shown prior to the start of the experiment.

### Statistical analysis

Analyses were performed using the statistical programming environment R. The Taylor expansion^26^ procedure was applied using the propagate package^27^ to calculate the expanded uncertainties associated with VOC measurements (Supplemental figure 2 Supplemental table1). The ratio of samples collected at 2.5, 5, and 7.5 ft were normalized by the volume-averaged concentration resulting in a series of magnifiers for each distance expressed in percentage values. The effect size associated with each magnifier was assessed using the Cohen’s D test.^28,29^

## Results

### Data normalization

Menthone, menthol, monoterpenes, isoprene, and acetone were selected for further analysis. We conducted paired t-test analyses between the first and last minute of baseline periods during which the participant did not consume breath mints in the chamber (n = 60). Table 2 presents the results of paired t-tests between the first and last minute of the baseline periods for each distance. The concentration of menthone, menthol, and monoterpenes did not change (p > 0.05) during baseline periods when the participant did not consume breath mints, while the concentration of isoprene and acetone changed during the baseline periods. This indicates that acetone and isoprene were detected in the participant’s natural breath. Upon further review, the changes were inconsistent with breath sources only, suggesting other indoor sources such as the participant’s skin and climate chamber interior materials may have contributed to the variability. Therefore, we summed the concentrations of menthone, menthol, and monoterpenes as unique breath tracer for the comparison of different distances in this study.

**Figure 2:**
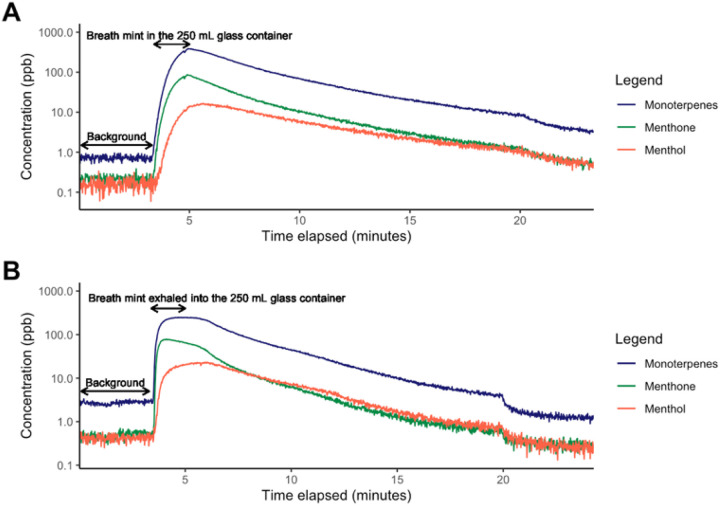
**A)** Concentration of three target tracer compounds (menthol, menthone, and monoterpenes) in the headspace of a 250 mL glass chamber as a function the time when a breath mint is placed inside, **B)** Concentration of the three target compounds when the participant exhaled their breath once into the 250 mL chamber while consuming the breath mint.

In addition to the analysis of baseline periods presented in Table 2, the presence of menthone, menthol, and monoterpenes in the exhaled breath of the participant while consuming breath mints was additionally confirmed through trials E & F (Table 1). Figure 2 indicates that the concentrations of menthone, menthol, and monoterpenes substantially increase when the breath mint was placed in the headspace of a 250 ml container (Figure 2A), or when the participant breathed naturally into the 250 ml container (Figure 2B).

Furthermore, Supplemental figure 1 shows the concentration of major VOCs during ventilation and baseline periods, indicating low but not necessarily zero concentrations for trials A-D (Table 1). We hypothesize that baseline concentrations are associated with residual VOCs that were adsorbed by climate chamber or ventilation filter surfaces and slowly re-emitted into the chamber air. To make a consistent starting point for all trials, we subtracted the average concentration of each compound detected during baseline periods from the 60-minute sampling period measurements.

**Figure 3:**
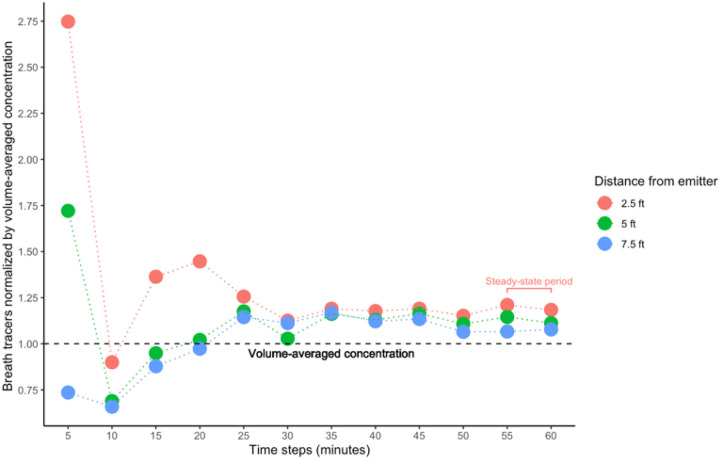
Comparison of 2.5, 5, and 7.5ft trials normalized by volume-averaged concentration.

The concentration of each compound for duplicate trials at each sampling location was averaged to produce a single data set for each of the distance trials (2.5 ft, 5 ft, 7.5 ft, and exhaust trials). Thereafter, the modified concentrations of menthone, monoterpenes, and menthol were summed to create a single value as breath tracer for each distance. A comparison of summed breath mint VOC concentrations normalized by the volume-averaged concentration is shown for each distance in Figure 3.

Supplemental table 1 reports on the magnifiers, expanded uncertainties associated with each value, as well as Cohen’s D effect size statistics for each distance. Supplemental figure 2 demonstrates the uncertainties associated with the values presented in Figure 3.

## Discussion

We used PTR-ToF-MS to trace the concentration of select VOCs associated with a consumed breath mint as a proxy for bioaerosol emissions from a healthy participant during each 60-minute trial. We summed the concentrations of menthone, monoterpenes, and menthol in each trial as a unique breath tracer since they were only detected only when the participant consumed breath mints. The summed tracer concentrations detected at 2.5, 5 ft, and 7.5 ft from the participant were normalized by volume-averaged concentration (VAC), which indicates the magnifier of each location compared with an approximate well-mixed condition. As shown in Figure 1, the concentration of VOCs at 2.5 and 5 ft rise above volume-averaged concentration during the first 5 minutes of the study, while the concentration at 7.5 ft begins to rise below the VAC level after minute 10 (Figure 2). We observed a steep increase in the concentration of breath tracers at 2.5, 5, and 7.5 ft during the first 5 minutes, which show that signals were first detected at closer range distances compared to the exhaust plenum due to a concentrated exhaled plume that had not mixed extensively throughout the chamber. At minute 5, the concentration of breath tracers also began to rise in the exhaust plenum, resulting in decreases in the ratios of indoor sampling locations normalized by the VAC (Figure 2). It took approximately 10 minutes for the concentration of breath tracers to become mixed in the chamber. At minute 10, the concentration of human tracers begins to increase in 2.5 ft trials against VAC, resulting in a higher concentration at 2.5 ft during minutes 5–20 compared to all other locations, with a 36–44% higher concentration than VAC. This finding suggests that the risk of exposure to virus-laden aerosol particles during the first 20 minutes is relatively higher for close contact distances (less than 3ft) when compared with other distances. Meanwhile, the concentration of breath mint tracers at 5 and 7.5 ft also rise above VAC during minutes 20–25, with the greatest magnifier having a value of 17% higher than VAC at 5 ft. After 25 minutes, tracer concentrations at 2.5, 5, and 7.5 ft maintained a relatively consistent trend in the ratio of distance-specific concentration normalized by VAC was observed. The magnifiers during this approximate steady-state period were ~18% (, ~11%(, and 7.5%(above VAC at 2.5, 5, and 7.5 ft, respectively. The expanded uncertainties associated with values reported in Supplemental table 1 are in agreement with previous studies that measured VOCs using PTR-ToF-MS.^30^ Despite the fact that Cohen’s d statistics show large effect size values when the concentration of breath tracers at 2.5, 5, and 7.5 ft were compared to VAC^31^, the uncertainty associated with our measurements suggest that reported magnifiers presented in Supplemental table 1 should be studied further with more replicates to improve the accuracy of and confidence in the near-field to far-field multipliers. Meanwhile, these findings highlight the importance of both near-field and far-field exposure events, and emphasize the importance of exposure duration in consideration of near-field and far-field prioritization. In this study, given these room characteristics and airflow rates the 20–25-minute event threshold helps to differentiate the dominance of near-field exposure risks, whereas longer events suggest dominance of far-field exposure minutes) risks.

Appendix A of the Supplemental document describes two case studies that present results of near- and far-field magnifiers for a recent controlled study on individuals that were diagnosed with COVID-19 (Supplemental table 2),^3^ as well as a study of patients undergoing high-flow nasal cannula therapy.^20^ Specifically, as shown in Supplemental tables 3, the concentration of CO_2_ and the particles of 1 μm – 2.5 μm in the controlled study on participants who were diagnosed with COVID-19^3^ were ~8 % and ~12 % higher in the near field (4 ft from the participants), compared to the far field (11 ft from the participants), respectively^3^. The present study provides confirmatory results in that the concentration of targeted VOCs in the near-field (2.5 ft) was ~10% higher than the far-field (7.5ft) during steady-state periods, thus-providing greater confidence for the concept of breath tracers as a proxy for virus laden bioaerosols.

## Conclusion And Limitations

Our study provides a series of magnifiers that could be used to estimate the concentration of bioaerosols at 2.5, 5, and 7.5ft from a human emitter in a reasonably well-mixed indoor space with ~ 3ACH over a 60-minute exposure event duration. These multipliers can be used in future studies of microbial risk assessment models to superimpose near-filed exposures and inhalation dose on far-field exposures estimated using a well-mixed assumption. Here we demonstrated that the concentration of detectable respiratory VOCs at 2.5, 5, and 7.5 ft were approximately ~18%, ~11%, and 7.5% higher, respectively, than volume-averaged concentration during steady-state periods. These magnifiers are reasonably consistent with values reported for a controlled study on participants diagnosed with COVID-19^3^ in which near-field (4 ft) was associated with 8% −17% higher concentrations for CO_2_ and particles (0.3 μm – 3 μm), resulting in 1 cycle threshold (C_T_), or two times, difference between the viral load detected in the near-field and far-field (Supplemental document, Appendix A).Our findings indicate that the concentration of bioaerosols in the far-field is relatively close to the well-mixed assumption at a steady-state period, while close range distances are associated with relatively higher exposure levels during the first 20 minutes of an emission exposure event.

Our study was a pilot project with several limitations. Our data were limited to replicated trials and a constant ventilation rate of ~3 ACH. There are several other environmental variables that can be studied through the same methodology such as the impact of relative humidity, temperature, mixing fan, facial masking, and room volume. Ventilation strategies other than overhead (used in this experiment) should be considered with different ACHs in future efforts. We seek to study several other distances & positions from the source emitter (vertical and horizonal distribution) to improve the accuracy of magnifiers with the intention of developing a comprehensive heterogeneous air space model for indoor air quality research.

## Supplementary Material

Supplement 1

## Figures and Tables

**Table 1.** Summary of all experiment trials

Trials	Sampling probe distance from the participant’s mouth	Number of replicates	Sampling frequency (Hz)	Sampling duration (minutes)	Number of samples
A	2.5 ft	2	1	60	3600
B	5 ft	2	1	60	3600
C	7.5 ft	2	1	60	3600
D	Exhaust	2	1	60	3600
E	Breath mint in a 250 ml glass container	1	1	20	1200
F	Breath mint exhaled into a 250 ml glass container	1	1	20	1200

**Table 2.** Comparison of the first and last minute of baseline period for five major compounds (paired t-test) for trials A-C (Table 1)

	Sampling distance from human source emitter (n = 60)		
Compounds	2.5ft	5ft	7.5ft
*Menthone*	0.0005(p = 0.92)	−0.005 (p = 0.32)	0.0107 (p = 0.1)
*Menthol*	0.0086(p = 0.46)	0.0189 (p = 0.35)	−0.0191 (p = 0.07)
*Mon oterpen es*	0.0015(p = 0.78)	0.0015 (p = 0.76)	−0.0015 (p = 0.79)
*Isoprene*	0.0704(p < 0.005)	−0.0556 (p < 0.001)	−0.04940 (p < 0.001)
*Acetone*	0.5982(p < 0.001)	−0.5913 (p < 0.001)	−0.3421 (p < 0.001)

## Synopsis

We offer a novel methodology for studying indoor air quality. We elaborated on near- and field-field exposures to virus laden particles, using VOCs as a proxy for bioaerosols emission.
