# Supplementary material for: A novel VOC breath tracer method to evaluate indoor respiratory exposures in the near- and far-fields
Source: Res Sq. 2022 Mar 11:rs.3.rs-1437107. Preprint. [Version 2] doi: 10.21203/rs.3.rs-1437107/v2 (PMC8923116; doi:10.21203/rs.3.rs-1437107/v2)
Supplement: Supplement 1 [file 4d722ad34ba48406ac062ac5.docx]

A novel VOC breath tracer method to evaluate indoor respiratory exposures in the near- and far-fields

Hooman Parhizkar^1,2^, Mark Fretz^1,2,3^, Aurélie Laguerre^4^, Jason Stenson^1,2^, Richard L. Corsi^5^, Kevin G. Van Den Wymelenberg^1,2,3*^, Elliott Gall^4^

1 - Institute for Health and the Built Environment, University of Oregon, Portland, OR, United States, 97209

2 - Energy Studies in Buildings Laboratory, University of Oregon, Eugene, OR, United States, 97403

3 - Biology and the Built Environment Center, University of Oregon, Eugene, OR, United States, 97403

4 - Department of Mechanical and Materials Engineering, Portland State University, Portland, OR, United States, 97201

5 - College of Engineering, UC Davis, Davis, CA, United States, 95616

***Corresponding Author:** Kevin G. Van Den Wymelenberg, kevinvdw@uoregon.edu, (541) 346-5647, Biology and the Built Environment Center, University of Oregon, Eugene, OR, United States, 97403, Energy Studies in Buildings Laboratory, University of Oregon, Eugene, OR, United States, 97403, Institute for Health and the Built Environment, University of Oregon, Portland, OR, United States, 97209

Supplemental figure 1 Evaluating the impact of distance on bioaerosol exposure in a typical indoor environment


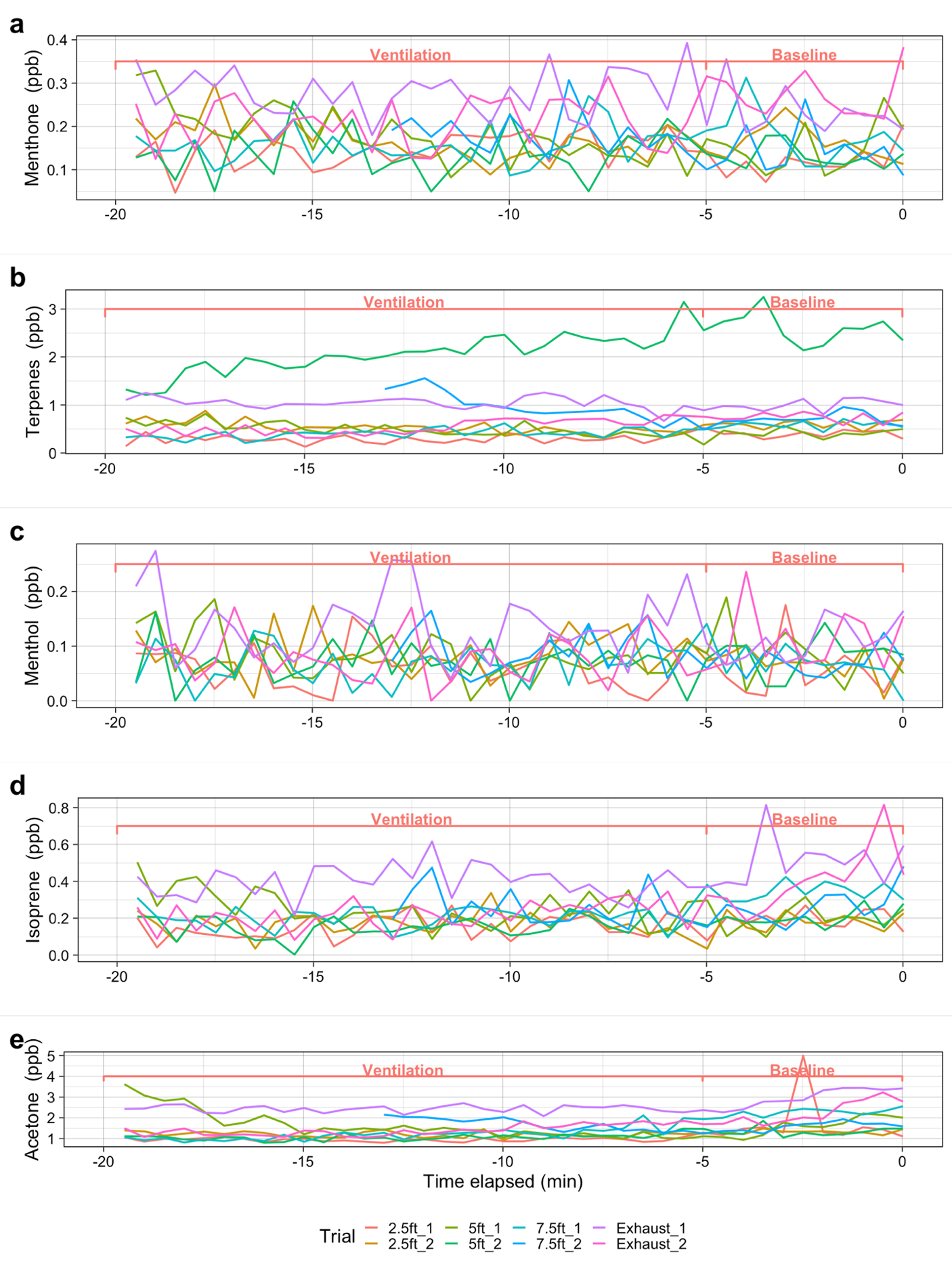


Supplemental table 1 Magnifiers and effect size values of 2.5, 5, and 7.5 ft normalized by volume averaged concentrations in 5 minutes time step

| 2.5 ft | | | 5 ft | | | 7.5 ft | | |  |
| --- | --- | --- | --- | --- | --- | --- | --- | --- | --- |
| Intervals | Magnifier | Effect size | $\pm$EU^2^ | Magnifier | Effect size | $\pm$EU^2^ | Magnifier | Effect size | $\pm$EU^2^ |
| Min_0_60 | 1.2123 | 0.32 (s) | 0.3496 | 1.0799 | 0.14 (N) | 0.3265 | 1.0516 | 0.1 (N) | 0.3094 |
| Min_0_5 | 2.7476 | 0.57(M) | 4.6822 | 1.7211 | -0.08(N) | 3.4974 | 0.7354 | -0.58(M) | 1.8898 |
| Min_5_10 | 0.8995 | -0.26(S) | 0.507 | 0.6893 | -0.95(L) | 0.51 | 0.6587 | -1.12(L) | 0.4337 |
| Min_10_15 | 1.3637 | 1.96(L) | 0.4228 | 0.949 | -0.42(S) | 0.3454 | 0.878 | -0.93(L) | 0.3048 |
| Min_15_20 | 1.4471 | 3.65(L) | 0.3764 | 1.021 | 0.09(N) | 0.3187 | 0.9731 | -0.28(S) | 0.2789 |
| Min_20_25 | 1.2559 | 2.00(L) | 0.3088 | 1.1754 | 1.32(L) | 0.2948 | 1.1446 | 1.07(L) | 0.2637 |
| Min_25_30 | 1.1251 | 1.39(L) | 0.2301 | 1.0277 | 0.23(S) | 0.2242 | 1.1122 | 1.08(L) | 0.2173 |
| Min_30_35 | 1.1901 | 2.13(L) | 0.251 | 1.162 | 1.78(L) | 0.2595 | 1.1689 | 2.12(L) | 0.2292 |
| Min_35_40 | 1.1774 | 2.00(L) | 0.2356 | 1.1293 | 1.35(L) | 0.2131 | 1.1219 | 1.41(L) | 0.2147 |
| Min_40_45 | 1.1904 | 2.54(L) | 0.2036 | 1.1633 | 2.27(L) | 0.2108 | 1.1343 | 1.88(L) | 0.1938 |
| Min_45_50 | 1.1518 | 2.39(L) | 0.1856 | 1.1082 | 1.71(L) | 0.1826 | 1.0647 | 1.01(L) | 0.1761 |
| Min_50_55 | 1.2103 | 2.76(L) | 0.2546 | 1.1452 | 2.01(L) | 0.217 | 1.0662 | 0.92(L) | 0.202 |
| Min_55_60^1^ | 1.1839 | 2.39(L) | 0.2559 | 1.1121 | 1.49(L) | 0.213 | 1.0775 | 1.03(L) | 0.1793 |

* N = negligible effect size, S = small effect size, M = medium effect size, L = large effect size

^1^ = Steady state period

^2^ =Expanded uncertainty

Supplemental figure 2. Comparison of the concentrations of breath tracers for each distance to volume integrated background with expanded uncertainties.


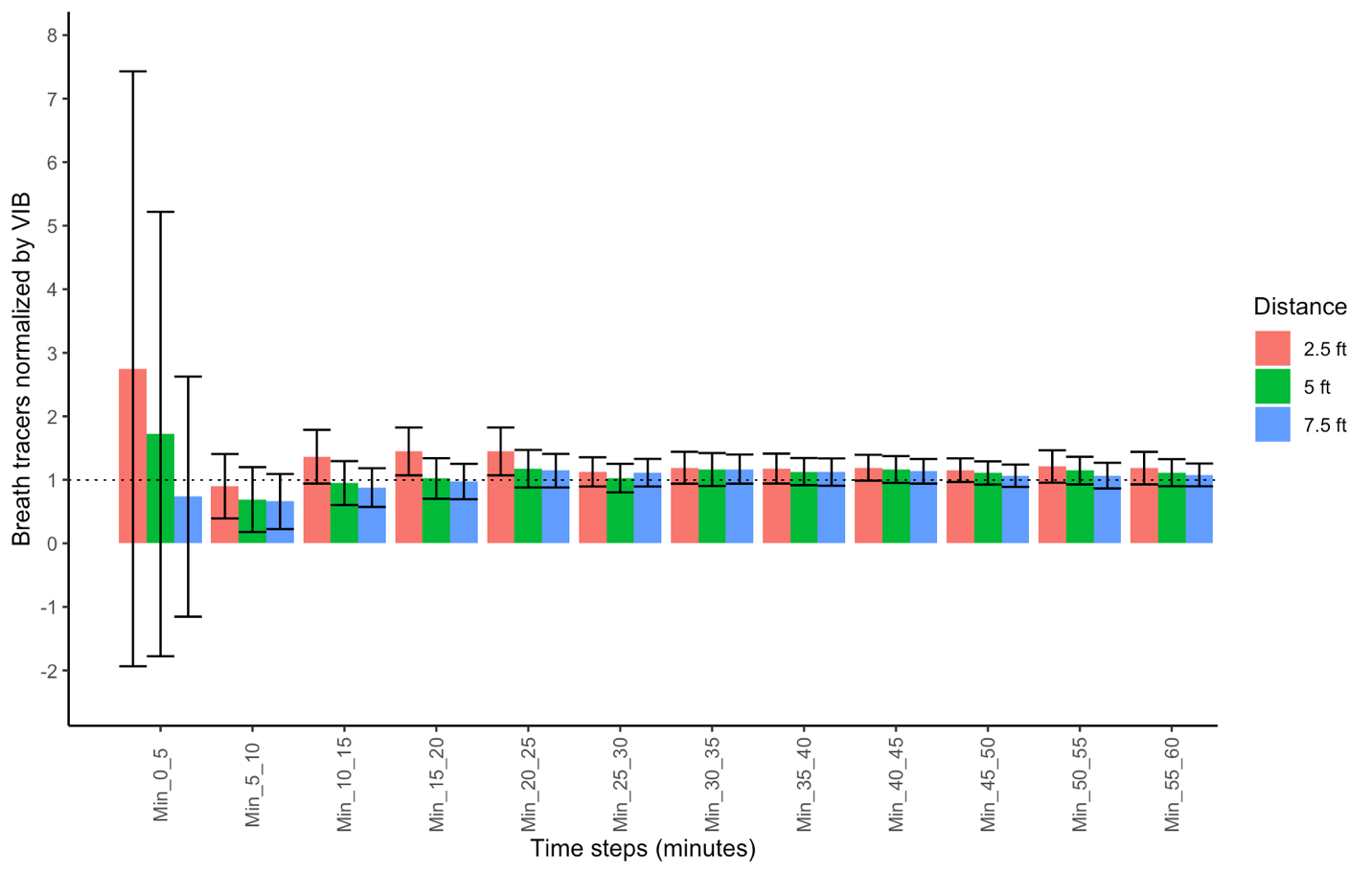


**APPENDIX A**

**The implications of magnifiers in a real-world case study**

The purpose of this section is to compare the results of near -field and far-field magnifiers for the present study and two recent relevant studies.^1,2^

In one study ^2^ the team measured near-field and far-field CO_2_ concentrations to estimate magnifiers in patient rooms within a healthcare environment having 8-11 ACH. The study reported background (far-field) CO_2_ levels of 580 ppm (mean across 7 patients) and reported near-field mean CO_2_ levels of 605 ppm, thus 25 ppm higher than background, which equates to a near-field magnifier of 4.3%^2^

A second study reports on bioaerosols emitted from individuals that were diagnosed with COVID-19 within a space having similar environmental conditions as the present study.^1^ Near-field and far-field terms were used to report the concentration of SARS-CoV-2 viral RNA in rom aerosols, room CO_2_, and room particles in the range of 0.3-25 µm at 1.2 m (4 ft) and 3.5 m (11 ft). We translate their data into near-/far-field ratios to provide a comparison with the near-field magnifiers reported in Figure 3 of the present study. The near-/far-field ratios from the previous study (Supplemental table 3) ranged from ~8-12% for CO2 and particles (1-2.5um), which correspond reasonably well with the near-field magnifiers of the present study (Figure 3) where the concentration of targeted VOCs in the near-field (2.5 ft) was ~10% higher than far-field (7.5ft) during steady-state periods.

Supplemental table 2 Comparison of spatial parameters between the present and the controlled study with participants diagnosed with COVID-19^1^.

| **Key variables** | **Breath tracer (present study)** | **Parhizkar, et al (2022)** |
| --- | --- | --- |
| **Volume (m3)** | 27 | 28.04 |
| **Air exchange rate (1/h)** | ~ 3 ACH | ~0 |
| **Duration (minutes)** | 60 | 60 |
| **Near- field distance (ft)** | 2.5 | 4 |
| **Far- field distance (ft)** | 7.5 | 11 |
| **Near/far fields ratio** | 3 | 2.75 |
| **Number of participants in the room for each trial** | 1 | 1 |

Supplemental table 3 Comparison of near- field and far-field in a recent controlled study on participants that were diagnosed with COVID-19.^1^

| **Variable** | *Near-field* | *Far-field* | **Near-field / Far-field** |
| --- | --- | --- | --- |
| CO2 (ppm) | 937.724 | 862.4149 | **1.0836** |
| Particles, 0.3 µm - 1µm | 16454.36 | 15959.25 | **1.0305** |
| Particles, 1 µm - 2.5 µm | 559.5155 | 493.7172 | **1.1249** |
| Particles, 2.5 µm - 3µm | 37.61776 | 31.71535 | **1.1702** |
| Particles, 3 µm - 5 µm | 50.8684 | 45.12782 | **1.1196** |
| Particles, 5 µm - 10 µm | 26.47086 | 27.24334 | N/A |
| Particles, 10 µm - 25 µm | 8.85414 | 8.961011 | N/A |

Reference

1. Parhizkar H, Dietz L, Olsen-Martinez A, et al. Quantifying environmental mitigation of aerosol viral load in a controlled chamber with participants diagnosed with COVID-19. *Clin Infect Dis*. January 2022. January 6, 2022. https://academic.oup.com/cid/advance-article/doi/10.1093/cid/ciac006/6498295. Accessed January 17, 2022.

2. Gall ET, Laguerre A, Noelck M, Van Meurs A, Austin JP, Foster BA. Near-field airborne particle concentrations in young children undergoing high-flow nasal cannula therapy: a pilot study. *J Hosp Infect*. 2021;113:14–21.
